# Letter to the editor: efficacy of different methods of combination regimen administrations including dexamethasone, intravenous immunoglobulin, and interferon-beta to treat critically ill COVID-19 patients: a structured summary of a study protocol for a randomized controlled trial

**DOI:** 10.1186/s13063-020-04499-5

**Published:** 2020-06-19

**Authors:** Nafiseh Abdolahi, Effat Kaheh, Roghieh Golsha, Behnaz Khodabakhshi, Alireza Norouzi, Mahmoud Khandashpoor, Sima Besharat, Samane Tavassoli, Somayeh Livani, Sadegh Ali Azimi, Mohammad Hadi Gharib, Babak Peivandi, Abdolreza Fazel, Hesamaddin Shirzad-Aski, Gholamreza Roshandel

**Affiliations:** 1 Golestan Rheumatology Research Center, Golestan University of Medical Sciences, Gorgan, Iran; 2 Infectious Diseases Research Center, Golestan University of Medical Sciences, Gorgan, Iran; 3 Golestan Research Center of Gastroenterology and Hepatology, Golestan University of Medical Sciences, Gorgan, Iran; 4 Clinical Research Development Center (CRDC), Sayad Shirazi Hospital, Golestan University of Medical Sciences, Gorgan, Iran; 5 Cancer Research Center, Golestan University of Medical Sciences, Gorgan, Iran; 6 Clinical Research Development Unit (CRDU), 5th Azar Hospital, Golestan University of Medical Sciences, Gorgan, Iran

**Keywords:** COVID-19, Randomised controlled trial, Protocol, Corticosteroids, Patients management, Inflammation

## Abstract

**Objectives:**

There is little information about Coronavirus Disease 2019 (COVID-19) management for critically ill patients. Most of these patients develop acute respiratory distress syndrome (ARDS) due to excessive inflammatory response and the ensuing cytokine storm. Anti-inflammatory drugs including corticosteroids can be used to effectively reduce the effect of this cytokine storm and lung damage. However, corticosteroids can have side effects, so simultaneous administration of immunoglobulin (IV-IG) and interferon-beta can help manage treatment using corticosteroids. Therefore, we designed a trial to test our hypothesis that early administration of dexamethasone in combination with IV-IG and interferon-beta can reduce the effect of the cytokine storm in critically ill patients COVID-19.

**Trial design:**

A phase two multi-center randomized controlled trial (RCT) with three parallel arms (1:1:1 ratio).

**Participants:**

They will be hospitalized patients with severe COVID-19 who have positive RT-PCR test and have blood oxygen saturation levels (SpO_2_) less than 90% and respiratory rate higher than 24 per minute or have involvement of more than 50% of their lung when viewed using computed tomography (CT)-scan. The age range of patients will be 18-70 years old.

Exclusion criteria: the need for intubation; allergy, intolerance, or contraindication to any study drug including dexamethasone, IV-IG, and interferon-beta; pregnancy or lactation; known HIV positive or active hepatitis B or C.

The study will be conducted in several hospitals of the Golestan province, Iran.

**Intervention and comparator:**

The study subjects will be randomly allocated to three treatment arms: two experimental groups (two arms: Intervention 1 and Intervention 2) and one Control Group, which will be matched for age and sex using frequency matching method. Each eligible patient in the control arm will receive the standard treatment for COVID-19 based on WHO guidelines and the Ministry of the Health and Medical Education (MOHME) of Iran. Each patient in the Intervention Group 1 will receive the standard treatment for COVID-19 and dexamethasone, at the first 24 hours’ time of admission. The intervention begins with the administration of dexamethasone based on the SpO_2_ levels. If the level of SpO_2_ does not improve after 24 hours, IV-IG (400 mg/kg once daily for 5 days) and interferon-beta (7 doses every other day) will be prescribed along with dexamethasone administration. In Intervention Group 2, the administration of dexamethasone will be started within the first 24 hours’ time of admission and will be continued for 48-72 hours and then the SpO_2_ level will be checked. Then, if the level of SpO_2_ has not improved after that time, IV-IG and interferon-beta will be prescribed as the same dosage as Group 1. If the percentages of the SpO_2_ level are between 85 and 90/ 80 and 85/ 75 and 80/ less than 75, the dosages will be 4 mg every 12 hours/ 4 mg every 8 hours/ 8 mg every 12 hours/ 8 mg every 8 hours, respectively.

According to the WHO recommendation, all participants will have the best available supportive care with full monitoring.

**Main outcomes:**

Primary: An increase in the SpO_2_ level to reach more than 90% in each case, which will be assessed by the oximeter.

Secondary: The duration of hospital stays; intubation status and the percentage of patients who are free of mechanical ventilation; the mortality rates during hospitalization and one month after the admission time.

**Randomisation:**

Participants will be allocated into either control or intervention groups with a 1:1:1 allocation ratio using a computer random number generator to generate a table of random numbers for simple randomization.

**Blinding (masking):**

The project's principal investigator (PI) is unblinded. However, the PI will not analyse the data and interpret the results. An unblinded researcher (a pharmacist) will cover the drug’s bottles with aluminium foil and prepare them interventions and control drugs in a syringe with a code so that patients are blinded. This person will have no patients contact. The staff and nurses, caring for the patients, will be unblinded for each study group due to the nature of this study. The staff that take outcome measurements will be blinded. The laboratory technicians will also be blinded as well as the statistical team. These study statisticians will have access to coded data and will analyse the data labelled as group X, group Y, and group Z.

**Numbers to be randomised (sample size):**

The target sample size will be 105 critically ill COVID-19 patients, who will be allocated randomly to the three trial arms with 35 patients in each group.

**Trial status:**

Recruitment is ongoing. The study began on April 18 2020 and will be completed June 19 2020. This summary describes protocol version 1; April 2 2020.

**Trial registration:**

https://www.irct.ir/. Identifier: IRCT20120225009124N4 version 1; Registration date: April 2 2020.

**Full protocol:**

The full protocol is attached as an additional file, accessible from the Trials website (Additional file 1). In the interest in expediting the dissemination of this material, the familiar formatting has been eliminated; this Letter serves as a summary of the key elements of the full protocol. The full protocol has been reported in accordance with the Standard Protocol Items: Recommendations for Clinical Interventional Trials (SPIRIT) guidelines.

## Supplementary information

**Additional file 1.**

## Data Availability

Chief investigators and the corresponding authors will have access to the final trial dataset. The corresponding authors will evaluate any request for data sharing and will consult with the steering committee after the publication of the main results. Requests can be sent to n_abdolahi2002@yahoo.com, roshandel_md@yahoo.com, and Shirzad_hessam@yahoo.com.

