# Supplementary material for: Letter to the editor: efficacy of different methods of combination regimen administrations including dexamethasone, intravenous immunoglobulin, and interferon-beta to treat critically ill COVID-19 patients: a structured summary of a study protocol for a randomized controlled trial
Source: Trials. 2020 Jun 19;21:549. doi: 10.1186/s13063-020-04499-5 (PMC7303932; doi:10.1186/s13063-020-04499-5)
Supplement: Supplementary file 1 — Additional file 1. [file 13063_2020_4499_MOESM1_ESM.doc]

Efficacy of Different Methods of Combination Regimen Administrations Including Dexamethasone, Intravenous Immunoglobulin, and Interferon-Beta to Treat critically ill COVID-19 Patients: Study Protocol for a randomized controlled trial

Nafiseh Abdolahi^a^, Effat Kaheh^a^, Roghieh Golsha^b^, Behnaz Khodabakhshi^b,c^, Alireza Norouzi^c^, Mahmoud Khandashpoor^d^, Sima Besharat^*b,c^, Samane Tavassoli^a^, Somayeh Livani^d^, Sadegh Ali Azimi^b^, Mohammad Hadi Gharib^a^, Babak Peivandi^d^, Abdolreza Fazel^e,f^, Hesamaddin Shirzad-Aski^*b^, Gholamreza Roshandel^*c^

1. Golestan Rheumatology Research Center, Golestan University of Medical Sciences, Gorgan, Iran
2. Infectious Diseases Research Center, Golestan University of Medical Sciences, Gorgan, Iran
3. Golestan Research Center of Gastroenterology and Hepatology, Golestan University of Medical Sciences, Gorgan, Iran
4. Clinical Research Development Center (CRDC), Sayad Shirazi Hospital, Golestan University of Medical Sciences, Gorgan, Iran
5. Cancer Research Center, Golestan University of Medical Sciences, Gorgan, Iran
6. Clinical Research Development Unit (CRDU), 5^th^ Azar Hospital, Golestan University of Medical Sciences, Gorgan, Iran

*co-corresponding authors:

Hesamaddin Shirzad-Ask, Ph.D., Infectious Diseases Research Center, Golestan University of Medical Sciences, Gorgan, Iran

Gholamreza Roshandel, Ph.D., Golestan Research Center of Gastroentrology and Hepatology, Golestan University of Medical Sciences, Gorgan, Iran;

E-mail: [Shirzad_hessam@yahoo.com](mailto:Shirzad_hessam@yahoo.com); roshandel_md@yahoo.com

Phone numbers: +989111701969; +989113757327

Fax: +981732251910

**Abstract**

**Background:** The pandemic of Coronavirus Disease 2019 (COVID-19) affects the whole health system and the global economy. One of the reasons is that there is unfortunately little information about disease management especially in critically ill patients of COVID-19. A significant percentage of these patients develop acute respiratory distress syndrome (ARDS) due to cytokine storm. We hypothesize early administration of corticosteroids alone or in combination with intravenous immunoglobulin (IV-IG) and interferon-beta can improve the outcome of the disease.

**Methods:** This is a phase two multi-center randomized controlled trial (RCT) with three parallel arms (1:1:1). In each arm, 35 subjects will be enrolled. Each eligible subject will be allocated in an intervention group or control group with a simple randomization method. The control and two other groups will be received the standard treatment for COVID-19. The intervention 1 group will be also received dexamethasone, at the first 24 hours' time of admission, and will be continued base on the improvement of the level of SpO_2_. Another group will be received dexamethasone for the first three days. In the groups of 1 and 2 interventions, IV-IG and interferon-beta will be supported the treatment after 24 or 72 hours of dexamethasone prescription, respectively. The cases will be followed up for one month after discharge. The primary outcome will be an improvement in the level of SpO2 of more than 90%. The secondary outcomes will be the hospital length of stay, intubation status, and the percentage of patients who are free of mechanical ventilation, the mortality rate during hospitalization, and one month after the admission time.

**Discussion:** There are many questions about the treatment and effectiveness of all used drugs in this COVID-19 situation. But, at present, there is no safety and effectiveness data from RCTs on corticosteroids in combination with IV-IG and interferon-beta against COVID-19. Cytokine storm and inflammatory mediators are two main suspects for ARDS in these patients. Theoretically, corticosteroids could help to reduce the incidence of ARDS and consequently the mortality rate. However, the aggregation of the results of different studies indicates an improvement in disease outcomes using corticosteroids in critically ill patients. This trial will try to show the effectiveness or disadvantages of corticosteroids, IV-IG, and interferon-beta administrations in critically ill COVID-19.

**Trial registration:** IRCT20120225009124N4 version 1

**Keywords:** Coronavirus; COVID-19; Randomized Controlled Trial; Protocol; Corticosteroids; Patients management; Inflammation

**Administrative information**

Note: the numbers in curly brackets in this protocol refer to SPIRIT checklist item numbers. The order of the items has been modified to group similar items (see <http://www.equator-network.org/reporting-guidelines/spirit-2013-statement-defining-standard-protocol-items-for-clinical-trials/>).

| Title {1} | Efficacy of Different Methods of Combination Regimen Administrations Including Dexamethasone, Intravenous Immunoglobulin, and Interferon-Beta for Treatment of Severe COVID-19 Patients: Study Protocol for a randomized controlled trial |
| --- | --- |
| Trial registration {2a and 2b}. | This trial was registered in the Iranian Registry of Clinical Trials (IRCT) system. This is a Primary Registry in the WHO Registry Network set up with help from the Ministry of Health and Medical Education (MOHME) and hosted by Iran University of Medical Sciences (IUMS).  Registry website: <https://www.irct.ir/> |
| Protocol version {3} | IRCT20120225009124N4 version 1; Registration date: April 2 2020. |
| Funding {4} | The Golestan University of Medical Sciences, Iran supported the present RCT (grant no. 111542). |
| Author details {5a} | Golestan Rheumatology Research Center, Golestan University of Medical Sciences, Gorgan, Iran; Infectious Diseases Research Center, Golestan University of Medical Sciences, Gorgan, Iran; Golestan Research Center of Gastroenterology and Hepatology, Golestan University of Medical Sciences, Gorgan, Iran; Clinical Research Development Center (CRDC), Sayad Shirazi Hospital, Golestan University of Medical Sciences, Gorgan, Iran; Cancer Research Center, Golestan University of Medical Sciences, Gorgan, Iran; AND Clinical Research Development Unit (CRDU), 5^th^ Azar Hospital, Golestan University of Medical Sciences, Gorgan, Iran. |
| Name and contact information for the trial sponsor {5b} | Name of sponsor organization is the Golestan University of Medical Sciences, Gorgan, Golestan, Iran.  The full name of the responsible person is Dr. Mohammadreza Honarvar.  Phone: +98 17 3245 1660; Email: info@goums.ac.ir |
| Role of sponsor {5c} | The funders had no role in the design of the trial, the intervention procedures, data collection, evaluation and analysis, and selection of a journal for submitting the final version of the present protocol. |

**Introduction**

**Background and rationale {6a}**

Coronavirus Disease 2019 (COVID-19) appeared in Wuhan, Hubei, China in December 2019 and the disease spread rapidly around the world since January 2020 and became a pandemic in March 2020. Severe acute respiratory syndrome coronavirus 2 (SARS-CoV-2) (formerly known as 2019-nCoV) is the etiology agent of the disease (1). The COVID-19 infected more than 6700000 individuals all around the world and more than 390000 of them have died, which makes an extraordinary time for our planet. The disease has put a lot of pressure on health care systems, because the number of patients is increasing exponentially, especially in the early days of the spread of infection in each area. Besides, there is no still vaccine or proven effective anti-viral therapy for the disease to help the clinicians to manage it. There is little information about disease management in critically ill patients. The World Health Organization (WHO) and the United States Center for Disease Control and Prevention (CDC) are still working and researching on the best guidance for the management of COVID-19 (2).

Currently, the Ministry of Health and Medical Education (MOHME) of Iran recommends a combination of different antiviral drugs including KALETRA (lopinavir and ritonavir), atazanavir and ritonavir with interferon-beta for the treatment of COVID-19 cases along with supportive therapy. However, this guideline emphasizes that the therapeutic effects of these drugs have not been conclusively proven [Available from: http://dme.behdasht.gov.ir].

Based on some evidence, a significant percentage of COVID-19 patients develop acute respiratory distress syndrome (ARDS). In this regard, the cytokine storm can be one of the most dangerous complications of the clinical onset (3). Furthermore, in the majority of these severe cases, similar to SARS, lymphopenia, and excessive inflammatory responses of the immune system have been reported, which are the main causes of lung tissue damage, and death of patients (4,5). Therefore, it seems that anti-inflammatory drugs including corticosteroids can be used to effectively reduce the effect of this cytokine storm in these patients and the rising mortality (3,4). Corticosteroids including dexamethasone can control the production and the amount of release of some cytokines including interleukin (IL)-1, IL-6, IL-8, IL-12, and Tumor necrosis factor-alpha (TNFα) (4,6). It should be noted that anti-inflammatory drugs including corticosteroids are not routinely recommended in viral infections, because they have several side effects and can reduce the power of the immune system and should be used with caution in people with the viral infections. However, some previous studies showed that corticosteroids can be helpful in critically ill patients with the hyper-inflammation situation in SARS-CoV (3,6,7).

Corticosteroids can be used with some other medications including intravenous immunoglobulin (IV-IG) to reduce the side effect of them and control the cytokine storm. IV-IG can regulate immune system activity. Results of various studies highlighted that IV-IG compensated the side effect of corticosteroids on the immune system (3,8). Interferon-beta is another factor that has anti-viral and anti-inflammation effects, which leads to being a drug candidate for COVID-19 (9).

Currently, the combinations of three categories of drugs including corticosteroids, IV-IG, and interferon-beta are used experimentally by clinicians in some centers of COVID-19 patients. in this regard, some unpublished data showed some effectiveness in the improvement of cases. However, the best time to start prescribing this regimen and the best candidate for patients to use these drugs are two of the main questions that are still unclear. In a study in China in 2020, Wang et al. reported that the administration of methylprednisolone with a dosage of 1-2 mg/kg/d for 5-7 days was effective in cases with severe COVID-19 pneumonia. These cases had a faster improvement of SPO_2_ and shorter hospital stay days compared to the control group (10). Moreover, in another study, two doses of dexamethasone were used in infected pigs with porcine respiratory coronavirus and the laboratory results suggested that the drug may effectively reduce the inflammatory response in the acute phase of infection (11). However, the results of the effectiveness of these drugs in different studies have been reported differently and have not yet been fully determined. The reason for this may be due to differences in the type and timing of medication. Due to this, we designed a trial to test a hypothesis that early administration of dexamethasone in combination with IV-IG and interferon-beta can reduce the effect of cytokine storm in the critically ill patient of COVID-19.

**Objective {7}**

The primary study objective is to analyse the efficacy of different methods of dexamethasone in combination with IV-IG and interferon-beta to treat critically ill COVID-19 patients.

**Trial design {8}**

This is a phase two randomized controlled trial (RCT) with three parallel arms (1:1:1 ratio). Participants will be recruited in three arms including control, intervention 1, and intervention 2 arms. The participants will be allocated in the study arms using a simple randomization method.

**Methods: Participants, interventions, and outcomes**

**Study setting {9}**

The study will be a multi-center study and conducted in hospitals of Golestan province, Iran. Qualified patients admitted to the hospitals will be included in the study. Data on demographic and risk factors will be collected using a questionnaire. Clinical data, as well as laboratory data and imaging results, will be collected from the hospital information system (HIS). Clinical data will be symptoms, systolic and diastolic blood pressure, chronic medical illnesses, and heart and respiratory rates. Important laboratory data will be complete blood count (CBC), coagulation factors, and blood chemistry characteristics.

**Eligibility criteria {10}**

The following eligibility criteria will be set for participants:

Inclusion criteria: age 18-70 years old; proven of severe COVID-19 cases.

Exclusion criteria: the need for intubation; allergy, intolerance, or contraindication to any study drug including dexamethasone, IV-IG, and interferon-beta; pregnancy or lactation; known HIV positivity or active hepatitis B or C.

A critically ill COVID-19 case is defined as a patient with positive RT-PCR test who have a blood oxygen saturation level (SpO_2_) less than 90% and respiratory rate higher than 24 per minute or have involvement of more than 50% of his/her lung when viewed using computed tomography (CT)-scan. Internal medicine, pulmonologist, and nurses will perform the interventions.

**Who will take informed consent?** **{26a}**

The potential trial participants will sign the informed consent form if they have decision-making capacity. In the situation of the participants are unable to fill out the form, an independent clinician will confirm in writing that the participant cannot fill out the form. Therefore, her/his authorized family will be informed of the study and will be told all the advantages and disadvantages of the study and they will have the free right to include their patient to the study. Whenever the patient gains his ability and consciousness to decide, she/he will be notified of the nature of the study and has the option to stay in the study or withdraw from the study. Based on the request of participants all collected data will be deleted.

**Additional consent provisions for collection and use of participant data and biological specimens {26b}**

There is no application to our trial.

**Interventions**

**Explanation for the choice of comparators {6b}**

Despite all the efforts of the international community, there is still no standard protocol for the treatment of COVID-19. High-quality RCTs can help to set up an effective treatment protocol for the disease. According to the WHO (12) recommendation, all participants will have the best available supportive care with full monitoring.

**Intervention description {11a}**

The study subjects will be randomly allocated in three treatment arms and divided into two experimental groups (two arms: intervention 1 and intervention 2) and one control group, which will be matched for age and sex using frequency matching method (Figure 1). Each eligible patient in the control arm will be received the standard treatment for COVID-19 (a combination of antiviral drugs) and the usual standard of care according to the protocol of MOHME of Iran. If the proposed standard treatment is changed, all patients will receive a new suggestion for further treatment. Each patient in the intervention 1 group will receive the standard treatment for COVID-19 and dexamethasone, (as described below), during the first 24 hours of admission. The dexamethasone will be administrated base on the level of SpO_2_. If the level of SpO_2_ does not improve after 24 hours, IV-IG and interferon-beta will be prescribed as shown below along with dexamethasone administration. In the intervention 2 group, the administration of dexamethasone will be started at the first 24 hours of admission and will be continued until 48-72 hours and the level of SpO_2_ will be checked. Then, if the level of SpO_2_ does not improve after that time, IV-IG and interferon-beta will be prescribed as the same.

Drug dosages and duration of the regimes are stated below for each intervention group. The intervention begins with the administration of dexamethasone:

If the percentage of the SpO_2_ level is between 85 and 90, the dosage will be 4 mg every 12 h,

If the percentage of the SpO_2_ level is between 80 and 85, the dosage will be 4 mg every 8 h,

If the percentage of the SpO_2_ level is between 75 and 80, the dosage will be 8 mg every 12 h,

If the percentage of the SpO_2_ level is less than 75, the dosage will be 8 mg every 8 h.

The doses of IV-IG will be 400 mg/kg once daily for 5 days and interferon-beta will be 7 doses every other day. As a note, before the IV-IG administration, the level of IgA will be checked.

**Criteria for discontinuing or modifying allocated interventions {11b}**

Participants can/will be withdrawn from the study if the patients or their families request to be excluded from the study or the study is thought to be dangerous for the patient or the risks are more than the potential benefits.

If signs of severe respiratory infections appear, treatment will be based on the doctor's opinion and the condition of each patient; as well as, the subject will be immediately withdrawn from the study. However, the patient's information will be collected in such unforeseen and adverse events and studied.

**Strategies to improve adherence to interventions {11c}**

All drugs will be masked for all blind partners and patients. People who prescribe the drugs and handle the patients are aware of the nature of the drugs. All patient sampling will be performed at specific times each day.

If one of the doses of the study’s drug is forgotten, the appropriate dose of that drug will be replaced in the next dose. The reason for forgetting or deleting the drugs is written to evaluate and reduce the amount of error.

**Relevant concomitant care permitted or prohibited during the trial {11d}**

The side effects of drugs will be severely examined daily and any possible treatment will be considered in advance. In some situations, and some patients, doctors may prescribe other drugs of dexamethasone family, such as methylprednisolone, with the equivalent dose. The standard regime for COVID-19, recommended by MOHME of Iran, may change in time during the study. Any changes in the medication regimen will be reviewed in a meeting with different clinicians and ways to use will be set up about further action.

**Provisions for post-trial care {30}**

The participants will be received the standard of care in all interventions and control arms during the study time and the days of follow up. The cases who suffer harm from trial participation, if any, will be received all kinds of free medical services.

**Outcomes {12}**

**Primary outcome**

An increase in the SpO_2_ level to reach more than 90% in each case, which will be assessed by the oximeter.

**Secondary outcomes**

The duration of hospital stays; intubation status and the percentage of patients who are free of mechanical ventilation; the mortality rates during hospitalization and one month after the admission time.

**Participant timeline {13}**

All participants will be visited daily as long as they are hospitalized. If a person is discharged from the hospital earlier than 14 days, all his/her outcome factors will be monitored by phone or visiting at home. The secondary outcomes will be checked on day 14 in hospitalized patients or on the day of discharge. Each participant will be followed for one month. All interventions will be administrated to patients at a specific time each day. Monitoring and sampling, if needed, will be taken at the same time for a case in each day.

Figure 1: Flow chart of randomization and treatment assignment


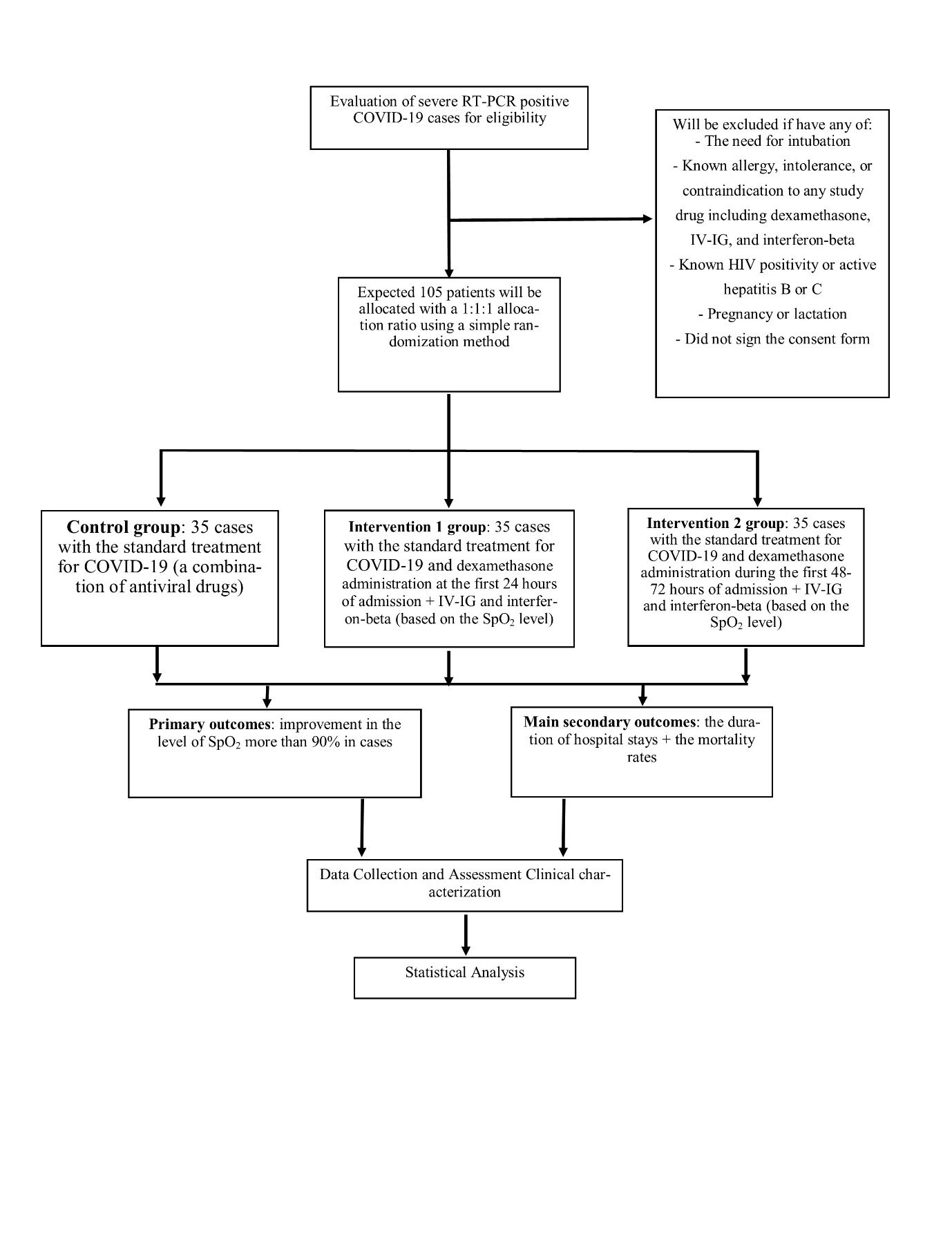


**Sample size {14}**

Based on the proposed effect size from previous studies (10) and assuming a two-sided 5% significance level and a power of 80%, 35 patients will be our sample size in each group. The target sample size will be 105 critically ill COVID-19 patients, which will be allocated randomly in three trial arms with 35 patients in each group. Patients will be allocated into study groups using a simple randomization method.

**Recruitment {15}**

On each day of the study, the main internal clinical specialist will examine all newly admitted patients in each morning round and select eligible cases based on the triage, laboratory, and CT-scan findings of the patients. One of the clinical colleagues of the project will put randomly patients in three arms and explain the study plan to the patient and provides the informed consent form. The project's lead executor (the staff member responsible for study) will review the availability of all the necessary facilities and make sure that the study is done properly.

**Assignment of interventions: allocation**

**Sequence generation {16a}**

Participants will be allocated into either control or intervention groups with a 1:1:1 allocation ratio using a simple randomization method (Figure 1). We will use a computer random number generator to generate a table of random numbers for simple randomization.

**Concealment mechanism {16b}**

The project's principal investigator (PI) (unblinded member) will do the randomization procedure and check the table of random numbers. The allocation process will be concealed to the physician and researchers. Allocation concealment will be made using an opaque sealed envelope system. For this purpose, the names of the groups will be placed in opaque closed envelopes, and whenever, according to the diagnosis of the physician, one of the patients will be eligible to enter the study, one of the envelopes will be randomly selected.

**Implementation {16c}**

The PI will do the randomization procedure. The main internal clinical specialist will screen all newly admitted patients in each morning round and selected eligible COVID-19 cases. A site investigator will explain the trial for each potentially eligible patient (or their family) and ask them to participate in the study. The site investigator and two research nurses will record the data of participants into patients’ medical charts and HIS. The project's lead executor will access to the data of participants. Two blinded research nurses will follow up on the visits or phone calls. The project's lead executor will send the data to the study therapist and statistical analyzer who are not involved in the study outcome. Each datasheet form will have randomization number codes. Staff responsible for recruitment and symptom ratings is not allowed to receive information about group allocation.

**Assignment of interventions: Blinding**

**Who will be blinded {17a}**

The PI is unblinded and is responsible for the study and will handle all team. However, this person will not play a role in examining the data and interpreting the results. An unblinded researcher (a pharmacist) will cover the drug’s bottles with aluminium foil and prepare them interventions and control drugs in a syringe with a code so that patients are blinded. This person will not be in contact with patients. The staff and nurses, caring for the patients, will be unblinded for each study group due to the nature of this study. The staff that take outcome measurements will be blinded. The laboratory technicians will also be blinded as well as the statistical team. These study statisticians will have access to coded data and will analyse the data labelled as group X, group Y, and group Z.

**Procedure for unblinding if needed {17b}**

If the main coordinator suspects about an adverse event in each patient, use the emergency treatment for the patient. In this regard, the coordinator should be discussed with the main staff member if he/she believes that unblinding is necessary. All procedures for unblinding will be documented and should be reported with reason. As a final note, discontinuation of treatment will not occur after the unblinding procedure.

**Data collection and management**

**Plans for assessment and collection of outcomes {18a}**

Several employees will daily complete all demographic and laboratory information of the patients and imported them to HIS. These persons are blinded about different study groups. The relevant patient data will be extracted via HIS and, if needed, will be completed by direct daily observation and review of patients' files by an unblinded person. The level of SpO_2_ (the primary outcome) will be daily measured by oximeter and the second outcomes will be extracted from HIS and evaluated. It will be assumed that all vital information of patients will be monitored continuously, as most data of patients in ICU are recorded as routine care.

**Plans to promote participant retention and complete follow-up {18b}**

All related data about the outcomes including the daily level of SpO_2_, vital status, intubation status, the hospital stay length, and the time of discharge will be obtained during the hospitalization, day 14, and followed up one month after the discharge time. The data will be obtained by HIS, physical visit on a bed or a telephone interview. The main coordinator will provide written feedback and send an acknowledgment to the participants or their families. Besides, all cases who withdraw their study participation will be followed one month and If they need management and health services, they will be taken care of. Moreover, information from people who have dropped out of the study will also be checked if they have received enough doses of the drugs and will not be replaced by other patients.

**Data management {19}**

All data will be entered into HIS. The system server is located in a secure data room and protected by the Golestan university firewall. Patient medical records (paper-based) will also be stored in a secure archive room and categorized with codes in numerical order. The backup files will be stored for five years. Only specific authorized people will have access to the files and access time will be recorded. The system output files will be sent as a password-protected Excel file using a flash drive for statistical review. A university VoIP (telephone) system will be set up to answer any participants’ possible questions in case of questions or problems.

**Confidentiality {27}**

The main coordinator will replace the name of each participant with a reference code in all electronically files including the password-protected Excel file. The main coordinator will store the main personal information of participants including their names and national ID numbers along with these reference codes in a secure cabinet throughout the study.

**Plans for collection, laboratory evaluation, and storage of biological specimens for genetic or molecular analysis in this trial/future use {33}**

We will not use any type of patients’ samples for future genetic or molecular analysis.

All plasma samples will be disposed of one week after the analysis due to the safety.

**Statistical methods**

**Statistical methods for primary and secondary outcomes {20a}**

All statistical analysis will be done by SPSS Statistics version 16 or will be analysed by R software. At first, the normality of variables will be checked using a Kolmogorov-Smirnov test. The continuous data with normal or skewed distribution will be analysed and showed using the number (N), mean and standard deviation (SD), or median and inter-quartile range (IQR), respectively. The frequencies and percentages will be presented for the other type of data including categorical data.

The effect of outcomes between two interventions and the control groups will be assessed by chi-square or Fisher’s exact tests for the categorical data and the independent *t*-test will be used to evaluate the normal distribution data.

**Interim analyses {21b}**

The interim analyses will be done after 15 patients enter in both intervention groups. After three unexpected serious adverse events in the intervention group leading to increase in the length of stay in the hospital, or one death event in both intervention groups, the main coordinator will check all procedures and report the results to the University committee, as well as he will make the final decision to terminate the trial for safety concerns.

**Methods for additional analyses (e.g. subgroup analyses) {20b}**

One of the researchers in our team will evaluate all data extracted from HIS and check the missing data. If possible, the subgroup analyses will be done based on the results.

**Methods in analysis to handle protocol non-adherence and any statistical methods to handle missing data {20c}**

Any missing data in the HIS information will be completed by a telephone interview, if possible. Protocol modifications and study subjects' replacement will not be expected. All primary and secondary outcomes, as well as adverse events, will be done as the intention-to-treat principle.

**Plans to give access to the full protocol, participant level-data and statistical code {31c}**

The full text of the protocol will be available after a formal and reasonable request to the corresponding author by email. Statistical data will be available 10 months after publication of the results in a scientific journal. The request for access to the de-identified patients' information will be reviewed by the team and will be accepted or rejected by reason.

**Oversight and monitoring**

**Composition of the coordinating center and trial steering committee {5d}**

The Coordinating Center (CC) will contain the main investigator who is an internal medicine specialist and an epidemiologist as a methodologist. These persons will report the result to the sponsor and the president of the Golestan University of Medical Sciences to use the result for the establishment of a new COVID-19 treatment algorithm. The trial steering committee will consist of the main investigator, internal specialists, infectious disease specialists, rheumatologists, radiologists, microbiologists, and study statisticians. The steering committee will supervise the trial and make a network for communication between the whole dependent and independent researchers and health workers. They will train everyone involved in the study.

**Composition of the data monitoring committee, its role and reporting structure {21a}**

A data monitoring team will evaluate the results, study safety, and data endpoints independently from the sponsor or any other groups. This team will contain one statistician and a clinician. The team will evaluate the data independently in three steps during the project progress and report only the final results to the sponsor.

**Adverse event reporting and harms {22}**

In case of any side effect or adverse event in each subject, a report will be written in detail and the main investigator will send the report to the steering committee of the study and the ethics committee of the University. For discharging patients, any complications associated with the study will be followed for one month. The main investigator will evaluate any life-threatening event leading to death. However, this randomized clinical trial will setup on severe COVID-19 patients who have a high mortality rate. For this reason, the cause of death of a patient will first be investigated and the causes will be reported. If it relates to the trail, the research will stop immediately.

**Frequency and plans for auditing trial conduct {23}**

An inspector team appointed by the ethics committee of Golestan University of Medical Sciences and on behalf of the national ethics committee will evaluate all procedures of the study. The main investigator will be independent of these inspectors as well as the sponsor and chief university managers.

**Plans for communicating important protocol amendments to relevant parties (e.g. trial participants, ethical committees) {25}**

Any changes to eligibility criteria, outcomes, analyses, and other important protocol modifications will be submitted in IRCT and they will review and approve them. Finally, the updated trial will be registered at [https://www.irct.ir](https://www.irct.ir/). The ethics committee will be aware of any changes and will approve them first.

**Dissemination plans {31a}**

The results will be published as an original article in a scientific journal after the completion of the study period and follow up on the cases.

**Discussion**

There are many questions about the treatment and effectiveness of used drugs in this COVID-19. Regarding the treatment of the disease, the issue of ARDS and Cytokine storm should be considered. ARDS acts as an important cause of death in critically ill patients of COVID-19. Cytokine storm and inflammatory mediators are two main suspects for this condition (3,4). All of these can lead to lung damage. Therefore, before extensive damage to lung tissue, clinical therapies should be considered on the control of the responses of the immune system and suppressing the inflammation, in critically ill patients. Theoretically, corticosteroids could help to reduce the incidence of ARDS and consequently the mortality rate (4). But their therapeutic effects and advantages and disadvantages are still in the mist.

Results of previous experiences in influenza and SARS and little new experiences in the management of COVID-19 are inconsistent, especially about the prescription time and number of corticosteroids in different phases of ARDS in the diseases. Use of corticosteroids in all cases of influenza or both SARSs may lead to secondary superinfections and increase mortality rate (12) and patients should be monitored very carefully and the medication should be given at the appropriate time. Besides, high-doses corticosteroids therapy may induce a worse outcome (13). However, the aggregation of the results of different studies indicates an improvement in disease outcomes using early doses of corticosteroids in critically ill patients. One systematic review evaluated the results of ARDS patient data from randomized trials and showed prolonged corticosteroid treatment improved clinical outcomes (14,15). Another randomized controlled trial study showed that corticosteroid therapy could down-regulate inflammation mediators in ARDS and reduce the duration of mechanical ventilation and staying in ICU (14). Surviving Sepsis Campaign has also recommended the use of corticosteroids if the patient is in the cytokine storm condition and if the patient has not yet developed ARDS (16). Some experiences from SARS showed that convalescent plasma or IV-IG can improve the survival rate of patients when they were treated with pulsed methylprednisolone. In this regard, we can use IV-IG to reduce the side effects of corticosteroids (15).

This trial has some limitations. This trial works on critically ill patients who have an inherently high mortality rate. This leads to loss to follow up and information or may affect the sample size. Secondly, due to the nature of the study, blinding is limited for some staff such as statisticians, and care providers cannot be blinded. Third, when we use allocation concealment using opaque envelopes, there is always a chance for subversion. Anyway, we will try to do fast this allocation to reduce the chance of subversion.

However, this trial is a multicenter RCT study and can provide more significant and useful data for the management of COVID-19. Generally, it is expected that this RCT show evidence that corticosteroids are effective in controlling cytokine storm and can use in the first stage of ARDS in critically ill COVID-19 cases.

**Trial status**

Recruitment Status: recruiting.

Protocol IRCT20120225009124N4 Version 1.

Registration date: April 2 2020.

Start of recruitment: April 18 2020.

End of recruitment (expected): June 19 2020.

**Abbreviations**

All abbreviations have been fully spelled when they first appear in the text.

**Declarations**

**Acknowledgments**

The authors thank all the nurses and medical staff who fight against COVID-19.

**Authors' contributions {31b}**

NA and GR are the chief investigators who conceived the study, led the project and protocol development, and made the final decisions; GR is study statistician and data analyst; SB and HS are a trial methodologist; AN is lead study coordinator; NA, EK, RG, BK, and ST contributed to study design and to development of the proposal. RG, BK, MK, SL, SAA, MHG, and AF collected the data, and evaluated and handled the patients and performed experiments, NA, GR, and HS drafted and wrote this version of the protocol. All authors read and approved the final protocol of the present RCT.

**Funding {4}**

The Golestan University of Medical Sciences, Iran supported the present RCT (grant no. 111542). The funders had no role in the design of the trial, the intervention procedures, data collection, evaluation and analysis, and selection of a journal for submitting the final version of the present protocol.

**Availability of data and materials {29}**

Chief investigators will have access to the final trial dataset. The corresponding authors will evaluate any request for data sharing and will consult with the steering committee after the publication of the main results.

**Ethics approval and consent to participate {24}**

The ethics committee of Golestan University of Medical Sciences approved the present trial (Ethics committee reference number: IR.GOUMS.REC.1399.004). All participants will freely complete the written informed consent form before entering the study.

**Consent for publication {32}**

Not applicable.

**Competing interests {28}**

The authors declare that they have no competing interests.

**Author information**

Nafiseh Abdolahi, Golestan Rheumatology Research Center, Golestan University of Medical Sciences, Gorgan, Iran, n_abdolahi2002@yahoo.com;

Effat Kaheh, Golestan Rheumatology Research Center, Golestan University of Medical Sciences, Gorgan, Iran, mayha56@yahoo.com;

Roghieh Golsha, Infectious Diseases Research Center, Golestan University of Medical Sciences, Gorgan, Iran, roghieh_golsha@yahoo.com;

Behnaz Khodabakhshi, Infectious Diseases Research Center, Golestan University of Medical Sciences, Gorgan, Iran, behkhoda@yahoo.com;

Alireza Norouzi, Golestan Research Center of Gastroenterology and Hepatology, Golestan University of Medical Sciences, Gorgan, Iran, norouzi54@gmail.com;

Mahmoud Khandashpoor, Clinical Research Development Center (CRDC), Sayad Shirazi Hospital, Golestan University of Medical Sciences, Gorgan, Iran, khandashpour2027@yahoo.com;

Sima Besharat, Golestan Research Center of Gastroenterology and Hepatology, Golestan University of Medical Sciences, Gorgan, Iran AND Infectious Diseases Research Center, Golestan University of Medical Sciences, Gorgan, Iran, s_besharat_gp@yahoo.com;

Samane Tavassoli, Golestan Rheumatology Research Center, Golestan University of Medical Sciences, Gorgan, Iran, tavassolisam54@gmail.com;

Somayeh Livani, Clinical Research Development Center (CRDC), Sayad Shirazi Hospital, Golestan University of Medical Sciences, Gorgan, Iran, dr_slivani@yahoo.com;

Sadegh Ali Azimi, Infectious Diseases Research Center, Golestan University of Medical Sciences, Gorgan, Iran, [ahaklksoldl@gmail.com](mailto:ahaklksoldl@gmail.com);

Mohammad Hadi Gharib, Golestan Rheumatology Research Center, Golestan University of Medical Sciences, Gorgan, Iran, hadigharib@yahoo.com;

Babak Peivandi, Clinical Research Development Center (CRDC), Sayad Shirazi Hospital, Golestan University of Medical Sciences, Gorgan, Iran, babak.peivandi1982@gmail.com;

Abdolreza Fazel, Cancer Research Center, Golestan University of Medical Sciences, Gorgan, Iran, AND Clinical Research Development Unit (CRDU), 5th Azar Hospital, Golestan University of Medical Sciences, Gorgan, Iran, fazelabdolreza@gmail.com;

Hesamaddin Shirzad-Aski, Infectious Diseases Research Center, Golestan University of Medical Sciences, Gorgan, Iran, shirzad.hessam1364@gmail.com;

Gholamreza Roshandel, Golestan Research Center of Gastroenterology and Hepatology, Golestan University of Medical Sciences, Gorgan, Iran, roshandel_md@yahoo.com;

**References**

1. Zhu N, Zhang D, Wang W, Li X, Yang B, Song J, et al. A Novel Coronavirus from Patients with Pneumonia in China, 2019. N Engl J Med [Internet]. 2020 Feb 20;382(8):727–33. Available from: http://www.nejm.org/doi/10.1056/NEJMoa2001017

2. World Health Organization. Coronavirus disease 2019 (COVID-19): situation report, 139. 2020;07 June 2020.

3. Mehta P, McAuley DF, Brown M, Sanchez E, Tattersall RS, Manson JJ. COVID-19: consider cytokine storm syndromes and immunosuppression. Lancet. 2020;395(10229):1033–4.

4. Azimi S, Sahebnasagh A, Sharifnia H, Najmeddin F. Corticosteroids Administration Following COVID-19-induced Acute Respiratory Distress Syndrome. Is it harmful or Life-saving? Adv J Emerg Med. 2020;4(2s):e43–e43.

5. Cheung CY, Poon LLM, Ng IHY, Luk W, Sia S-F, Wu MHS, et al. Cytokine responses in severe acute respiratory syndrome coronavirus-infected macrophages in vitro: possible relevance to pathogenesis. J Virol. 2005;79(12):7819–26.

6. Balzarini J. Carbohydrate-binding agents: a potential future cornerstone for the chemotherapy of enveloped viruses? Antivir Chem Chemother. 2007;18(1):1–11.

7. Russell B, Moss C, George G, Santaolalla A, Cope A, Papa S, et al. Associations between immune-suppressive and stimulating drugs and novel COVID-19—a systematic review of current evidence. Ecancermedicalscience. 2020;14.

8. Arumugham VB, Rayi A. Intravenous Immunoglobulin (IVIG). In: StatPearls [Internet]. StatPearls Publishing; 2020.

9. Abdolvahab MH, Mofrad MRK, Schellekens H. Interferon beta: from molecular level to therapeutic effects. In: International review of cell and molecular biology. Elsevier; 2016. p. 343–72.

10. Wang Y, Jiang W, He Q, Wang C, Wang B, Zhou P, et al. Early, low-dose and short-term application of corticosteroid treatment in patients with severe COVID-19 pneumonia: single-center experience from Wuhan, China. medRxiv. 2020;

11. Zhang X, Alekseev K, Jung K, Vlasova A, Hadya N, Saif LJ. Cytokine responses in porcine respiratory coronavirus-infected pigs treated with corticosteroids as a model for severe acute respiratory syndrome. J Virol. 2008;82(9):4420–8.

12. World Health Organization. Clinical management of severe acute respiratory infection (SARI) when COVID-19 disease is suspected: interim guidance, 13 March 2020. World Health Organization; 2020.

13. Takaki M, Ichikado K, Kawamura K, Gushima Y, Suga M. The negative effect of initial high-dose methylprednisolone and tapering regimen for acute respiratory distress syndrome: a retrospective propensity matched cohort study. Crit Care. 2017;21(1):135.

14. Meduri GU, Bridges L, Shih M-C, Marik PE, Siemieniuk RAC, Kocak M. Prolonged glucocorticoid treatment is associated with improved ARDS outcomes: analysis of individual patients’ data from four randomized trials and trial-level meta-analysis of the updated literature. Intensive Care Med. 2016;42(5):829–40.

15. Veronese N, Demurtas J, Yang L, Tonelli R, Barbagallo M, Lopalco P, et al. Use of Corticosteroids in Coronavirus Disease 2019 Pneumonia: A Systematic Review of the Literature. Front Med. 2020;7:170.

16. Alhazzani W, Møller MH, Arabi YM, Loeb M, Gong MN, Fan E, et al. Surviving Sepsis Campaign: guidelines on the management of critically ill adults with Coronavirus Disease 2019 (COVID-19). Intensive Care Med. 2020;1–34.
